# Supplementary material for: Content-rich biological network constructed by mining PubMed abstracts
Source: BMC Bioinformatics. 2004 Oct 8;5:147. doi: 10.1186/1471-2105-5-147 (PMC528731; doi:10.1186/1471-2105-5-147)
Supplement: Additional File 2 — The original results of the above study (non-essential files are deleted to keep the file size under the limit set by BMC bioinformatics). [file 1471-2105-5-147-S2.bz2 › chilibotAdditionalFile2/dip05/409ID10212223E10070/html/PTPRA_MDK.html]

 


 **PTPRA** and **MDK** 
  
Found 3 abstracts in PubMed, retrieved 3.  
 

 What does Google say? 
 PDF only 
| .edu only 

---

**Interactive relationship** (e.g. stimulation, inhibition, etc)

**Neutral relationship**- ... transfection of an LRP  [ **PTPRA** ]  expression vector restored midkine  [ **MDK** ]  internalization and subsequent nuclear translocation.  Ref: 12215536 Mol Cell Biol, 2002
- Here we demonstrate that LRP  [ **PTPRA** ]  mediates nuclear targeting by midkine  [ **MDK** ] .  Ref: 12215536 Mol Cell Biol, 2002
- We recently showed that LRP  [ **PTPRA** ]  binds heparin binding growth factor midkine  [ **MDK** ] .  Ref: 12215536 Mol Cell Biol, 2002

**Non-interactive relationship** (e.g. studied together, co-existance, homology, etc.)

- Midkine  [ **MDK** ]  was not internalized in LRP  [ **PTPRA** ]  deficient cells.  Ref: 12215536 Mol Cell Biol, 2002
